# Time Perception Test in IntelliCage System for Preclinical Study: Linking Depression and Serotonergic Modulation

**DOI:** 10.3390/diagnostics15020151

**Published:** 2025-01-10

**Authors:** Olga Sysoeva, Rauf Akhmirov, Maria Zaichenko, Ivan Lazarenko, Anastasiya Rebik, Nadezhda Broshevitskaja, Inna Midzyanovskaya, Kirill Smirnov

**Affiliations:** 1 Institute of Higher Nervous Activity and Neurophysiology, Russian Academy of Sciences, 117465 Moscow, Russia; akhmir5000@gmail.com (R.A.); mariya-zajchenko@yandex.ru (M.Z.); lazarenko_ia@ihna.ru (I.L.); rebik_anastasiya@mail.ru (A.R.); multibroshka@mail.ru (N.B.); midzyanovksya.is@ihna.ru (I.M.); smirnov.kirill.sc@gmail.com (K.S.); 2 Center for Cognitive Sciences, Sirius University of Science and Technology, Olimpiyskiy Ave. b.1, 354340 Sirius, Russia

**Keywords:** interval timing, depression, animal model, IntelliCage, serotonin reuptake inhibitors (SRIs)

## Abstract

**Background/Objectives::** The link between serotonergic modulation and depression is under debate; however, serotonin reuptake inhibitors (SRIs) are still the first-choice medicine in this condition. Disturbances in time perception are also reported in depression with one of the behavioral schedules used to study interval timing, differential-reinforcement-learning-of-low-rate, having been shown to have high predictive validity for an antidepressant effect. Here, we introduce an IntelliCage research protocol of an interval bisection task that allows more ecologically valid and less time-consuming rodent examination and provides an example of its use to confirm the previously reported acute effect of an SRI, clomipramine, on interval timing (increase in bisection point, D50). **Methods:** Wistar male rats (*n* = 25, five groups of 5–8) were trained in the IntelliCage to discriminate between short (1 s) and long (4 s) LED light stimuli by nose poking at the corresponding (left/right) side of the IntelliCage chamber to obtain a drink. When 80% of correct responses were reached, the intermediate durations of 1.7, 2.5, and 3.3 s were introduced. The number of left/right choices for each stimulus and interval timing parameters (bisection point, D50, and timing precision), derived from them, were compared after saline and clomipramine (7 mg/kg, i.p) intraperitoneal administration. **Results**: Rats successfully learned the task within about a week of training. The slightly increased D50 after clomipramine confirmed previous studies. **Conclusions:** The introduced protocol has potential to be applicable to preclinical research on depression and potentially other psychopathology, where time perception can be disturbed.

## 1. Introduction

Time perception is a basic cognitive function allowing organisms to effectively adapt to the predicted environment. At the same time, alterations in time perception are seen in several psychopathological conditions, such as depression [1,2]. Previous studies suggested a significant involvement of the serotonergic system in interval timing and time perception both in animals and humans [3,4,5,6]. The link between depression and lower serotonergic activity was also established a long time ago [7] and receives support from recent research (e.g., [8,9]). While the mechanistic explanation of this link is under debate [10], serotonin reuptake inhibitors (SRIs) are still the most used and efficient treatment of this condition [11].

Our previous study [12] of patients with Major Depressive Disorder (MDD) linked depression both to serotonergic hypofunctioning, assessed by neurophysiological correlates of central serotonergic functioning [13,14,15,16,17], and disturbance of time perception. To assess individual characteristics of time perception in this study, we used a two-alternative forced-choice duration discrimination task where participants had to compare two durations from about a 3 to 6 s duration range that covers genuine time experience [18,19,20]. Relative shrinkage of the first interval in relation to the second is typically observed in this task, corresponding to the temporal order effect [21,22,23]. This effect was less evident in patients with MDD, who experienced less subjective shortening of the duration, or in terms of psychophysical parameters, a higher point of the subjective equality (PSE) point, where first and second durations were perceived as similar. This effect can be mechanistically explained in terms of the lossy integrator model for time [22,24,25], which assumes continuous inflow and outflow of “duration/time”. We chose it as the only model that can explain the temporal order effect and also as more biologically plausible (no need to emit discrete pulses and then to count them, etc.). Previous studies also linked the relaxation rate of this lossy neuronal accumulator to activity of the serotonergic system with a higher loss rate associated with greater serotonergic modulations and at the behavioral level, reflected as more subjective shortening of the first stimulus’s duration compared to the duration of a second stimuli in a duration discrimination task, as well as a more pronounced underreproduction of the duration in a duration reproduction task in humans [4,5,26].

In the current report, we aim to link these findings to those obtained in animals. While there are few behavioral tests to infer about time perception in animals, e.g., a free-operant timing schedule or fixed-interval peak procedure (see recent systematic review [6]), most similar to the duration discrimination task used in our research with patients is an interval bisection task (IBT). In this task, animals are trained to respond to a specific pedal depending on the duration of stimuli (long vs. short). In the testing session, additional stimuli of intermediate durations are introduced and the probability of the response associated with the long/short pedal is recorded. Based on these measures, the psychometric curve is fitted and the interval bisection point (D50), where animals choose long/short responses with equal probability, is estimated. This value seems to correspond well with the PSE, derived from human studies in the duration discrimination task; however, the effects of drug treatment should be opposite due to the fact that in humans, both stimuli to compare are introduced within seconds and in the same functional condition of the participant. For animals, the reference point for comparison is usually established under the baseline neutral condition, but the effect of treatment is measured under the treatment. Thus, if serotonergic activation increases the loss rate of a putative temporal accumulator, then animals under increased serotonin activation (e.g., under clomipramine treatment) should have D50 shifted to the right (increased) as the duration should be objectively longer to be perceived as comparable to the representation for the long stimuli formed under the baseline condition.

Indeed, that is what has been shown by previous studies in animals. In IBT, the D50 was increased by agents that increase serotonergic activity, e.g., an SRI [27] or the 5-HT2A receptor agonists, such as DOI and quipazine [28,29,30]. Noteworthily, effects of agonists were counteracted by 5-HT2A receptor antagonist ketanserin and MDL-100907, but not 5-HT3 agonist m-CPBG, suggesting a primary role of 5-HT2A receptors in this process [28,29]. A decrease in D50 was found when an SB-242084 agent that blocked 5-HT2C was applied to mice [31,32], pointing to the implication of 5-HT2C receptors as well. While different targets within the serotonergic system seem to be involved (5-HT transporter, 5-HT2A and 5-HT2C receptors) in interval timing, all mentioned studies converge on the link between increased serotonergic neurotransmission and increased D50 that we relate to increased internal time speed through the lossy integrator model for time.

Notably, one of the widely used behavioral tests in preclinical trials of depression is the differential reinforcement of a low rate (DRL) schedule [33,34,35,36]. During the task, animals are reinforced if their response time is larger than a particular duration usually ranging from 10 to 72 s. DRL showed high predictive validity for an antidepressant effect. In particular, an SRI shifts the location of the peak interresponse interval to the right, indicating the ability to withhold a response for a longer time [33,34,37,38]. While several cognitive functions are involved in the performance of the DRL task, the recent studies support the crucial role of the timing process [39,40]. Moreover, the increase in the location of interresponse peak time can also be interpreted within the lossy temporal integrator model. Using similar logic to the logic we applied for IBT, an increased location of interresponse peak time corresponds with an increased loss rate of the putative temporal accumulator and thus suggests an increase in serotonergic modulation that is expected from SRI drugs. A similar effect is observed when treated with a psilocybin agonist of 5HT2A receptors [34]. Thus, cumulative evidence from this test also suggests that the modulation of serotonergic activity affects interval timing in DRL in a similar manner to that in the IBT.

While the relation between serotonergic modulation and interval timing in DRL and IBT is similar and can be interpreted within the same framework of the lossy temporal accumulator, we have found only one previous study that examined the effect of an SRI in IBT, the study by Bizot [27]. We believe that it is an unfortunate oversight as performance in IBT might also provide valuable information for preclinical studies. In this brief report, we aim to introduce IBT adapted to an ecologically valid automatized IntelliCage system [41,42,43,44], as well as to replicate the effect of SRI antidepressant clomipramine on Wistar rats’ performance in this test. In particular, we examine the hypothesis if the D50 is increased after acute treatment with clomipramine as can be derived from Bizot [27]. We assume that the investigation of the nuanced effect of an SRI on the cognitive functions, also associated with depression, such as interval timing, is of high importance as it can allow more focused understanding of its work.

## 2. Materials and Methods

A total of 29 male Wistar rats aged approximately 4–8 months and weighing 300–450 g at the start of the experiment were housed in five groups of 5–8 animals at a constant cycle of 12 h light and 12 h darkness (lights on: 04.00–16.00 h) at a temperature of 25 ± 1 °C. A month prior to the main experiment, all rats were implanted with individual identification veterinary chips. The chips were implanted subcutaneously above the neck muscles under mild sedation using dexmedetomidine (0.2 mg/kg, intramuscularly). Following chip implantation, the effect of dexmedetomidine was reversed by administering atipamezole (1 mg/kg, intramuscularly) to ensure rapid recovery. Animals were returned to their home cages only after full recovery from sedation, with constant monitoring to confirm normal behavior and mobility. Three rats were excluded from the experiment because the IntelliCage unit did not recognize the implanted chips, so drinking activity was not recorded in these animals. One rat died during the experiments for an unknown reason. Twenty-five rats completed the IntelliCage procedure but the psychometric function could be successfully fitted in the clomipramine condition only in twenty-two of them (see analysis for details).

### 2.1. Apparatus

The IntelliCage (TSE Systems GmbH, Berlin, Germany; version of 2011 with modification in 2016) is an automated group-housing apparatus allowing experimental testing within the home cage. The cage (410 × 190 × 435 cm) is equipped with four operant conditioning chambers located in each corner. Only three corners were used in our study, due to technical issues. Each conditioning chamber contains two water-drinking bottles and an LED light bulb that is switched on and off for particular durations and serves as a stimulus. Chambers are accessible by a small tunnel containing a transponder reader antenna that registers the microchip of the entering rat. Access to each water bottle is controlled by gated nose-poke holes containing infrared beam-break sensors, which can be programmed to open or remain closed upon a visit or nose-poke response. As each rat was implanted with a unique microchip, corner entry and nose-poke data could be integrated with microchip readings collected by each conditioning corner’s antenna, allowing data to be separated by each individual rat.

### 2.2. Experimental Procedure

For the first 2–7 days, animals were adapted to the behavioral chamber with free access to food and water. During the following week, animals could access water after poking with their nose, either through the right or left doors, separating the aperture space from the drinker. To adapt the animals to the new light regime, the onset of the light phase was shifted from 08:00–20:00 to 04:00–16:00 every two days during the week in 2 h increments per shift.

The next was the training phase in which 1 s and 4 s LED light turned on immediately as the rat entered the chamber. The presentation of a short stimulus (1 s LED) allowed access to water as a result of nose poking to the left side, and the presentation of a long stimulus (4 s LED light) allowed access to water as a result of nose poking to the right side. All responses before the LED light offset were considered premature, but did not stop the trial. All responses after the LED light offset were characterized as timed. Presentation accuracy was ±100 ms. This configuration was persistent for all groups. Stimuli were presented in random order with equal probability. However, if during the training, the animal responded incorrectly, this duration was presented again until a correct response was made. If the animal did not respond, the trial was considered complete without a significant nose poke encountered. This is called an omission trial. Our study design did not incorporate the auditory signal, but some sounds might be initiated by touching.

The training phase was carried out until the percentage of errors (choosing the wrong side) in the animals was reduced to 20%. For the first group of rats, however, this percentage did not separate responses by stimulus and it could be that a rat made more than 20% errors for one type of stimulus (prolonged) and less than 10% for another.

**Figure 1:**
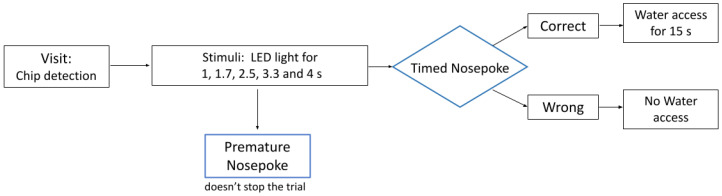
A schematic representation of the experimental trial in the IntelliCage behavioral protocol testing.

An experimental phase was then conducted for a week in which, in addition to long and short durations, intermediate durations (1.65, 2.5, 3.25 s) were presented that were not reinforced with water. The probability of the presentation of each stimulus (1, 1.65, 2.5, 3.25, and 4 s) was 20%. For intervals of 1 and 4 s, if the choice was correct, the side continued to be reinforced with water. If the choice was made incorrectly, just as in the training series, the presentation of this stimulus was repeated until the correct response was made. The schematic representation of the experimental trial within implicated protocol can be seen at Figure 1.

### 2.3. Drug Treatment

Treatments were given intraperitoneally (1 mL/kg of body weight) using a 27-gauge needle with switching between sides after each injection. Injections started just after the training phase (for one group of 5 rats) or after a week adaptation with an experimental phase that included intermediate durations (for 4 other groups). In the final analysis, all animals were united and considered irrespective of the group. Drug administration was performed approximately 30–45 min before the onset of the dark phase (16.00). Control conditions included three days of saline solution injections (1 mL/kg, i.p.), after which the phase with the administration of the pharmacological drug began, in which animals were injected with clomipramine (by FSUE SPC “Farmzashita”, 7 mg/kg, i.p.) every other day. For the current analysis, we took only the first 8 h after the first three injections.

### 2.4. Analysis

A Python-based program (scipy and numpy libraries were used) code was assembled to process the data, allowing filtering and sorting of events of interest. Activity recorded between 16:00 and 24:00 of the day of injection was analyzed. This period was chosen as a period during which the animals had increased drinking activity due to the onset of the dark phase and during which acute exposure to pharma drugs was observed, according to the previous literature [45,46].

For the current analysis to receive enough data for assessing probability of responses to each duration, we united data from three days. The number of nose pokes to the right/left bottle after the presentation of each duration was extracted for each animal. To assess total activity, these values were summed up. Premature responses (before the offset of LED light) and omission trials (a visit to the chamber without a significant nose poke) were not included in this total activity of productive visits, but assessed separately.

The parameters of the psychometric function were determined by the nonlinear least squares method. Experimental data (five combinations of t and P(long|t), for both saline and clomipramine) were used to find individual parameters for each rat.

P(long|t) = Φ{c (t − D50)/(ε)}

where P is the probability of choosing the “long/right” corner/bottle for a particular presented duration, t is the duration of presented stimuli, Φ is the cumulative normal density function, D50 is the stimulus duration corresponding to P(long|t) = 0.5 (also known as interval bisection point), ε is a variable inversely proportional to the slope of the function, and c is the constant equal to 0.6745 (the 75% quantile of the normal distribution). The slope of the function was defined separately by calculating the maximum value of the derivative of the obtained function at [0, 4]. It was experimentally confirmed that the P value at the point of the maximum derivative never differed from 0.5 by more than 0.01.

In four cases that correspond to three rats (for one rat in both conditions and for two rats only in clomipramine condition), it was not possible to find a logistic curve with realistically achievable parameters (e.g., D50 >> 4, ε >> 2), or the required number of iterations exceeded 10,000. These three rats were removed from the sample. Moreover, they also had low total activity, e.g., less than 10 visits per the 1 or 4 s condition, making the measures of probability assessment within the chosen interval unreliable. Noteworthy, none of the other rats had such a low number of visits per these conditions.

The Shapiro–Wilk test was used to examine the distribution of the obtained values on normality. If the data were normally distributed, the parametric Student’s *t*-test was used. When data distribution deviated from normality, we employed the non-parametric paired Wilcoxon signed rank test. The following parameters were compared: D50, slope, total activity, omissions, and premature nose-poke numbers in the saline and clomipramine conditions. As we had a particular hypothesis to examine for the D50 shift, e.g., the increase in D50 after clomipramine, we employed the one-tailed Student’s *t*-test. As no particular expectations existed for the other parameters examined, a two-tailed test was applied. Hedge g was used to measure the effect size. Spearman correlations were used to examine the relation on the effect of clomipramine on different parameters.

## 3. Results

Testing interval timing within the IntelliCage system proved to be successful and efficient. All rats could reach a predefined learning level of less than 20% of errors within 7–10 days. For the further analysis, we took the total number of timed nose pokes (total activity), omissions (trials with no nose pokes), and premature nose pokes (nose poke during light presentation) for the 8 h interval after injection across three injection days of either saline or clomipramine. The D50 and slope values were derived from a psychometric function fitted into the probability of right/long corner/bottle choices for each duration also calculated for the same period. The Shapiro–Wilk test confirmed normal distribution for all our studied parameters except for the number of premature responses.

**Figure 2:**
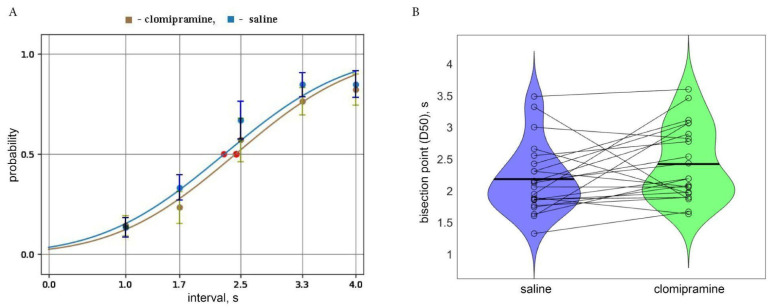
(**A**). The psychometric curve obtained in the saline (blue) and clomipramine (green) conditions with corresponding probability measures (mean and std) averaged across all rats included in the analysis (*n* = 22). Red dots correspond to bisection points (D50) in both conditions. (**B**) Violin plots of D50 in the saline and clomipramine conditions. Horizontal lines correspond to mean group values. Each dot corresponds to individual values obtained for each rat and the lines connect the points of the same rat in two conditions.

Figure 2A represents the psychometric curve average across all rats, included in the analysis (*n* = 22). The parameters of D50 and ε were averaged across all studied rats (*n* = 22). Acute clomipramine treatment led to an increase in D50 compared to the saline condition (t(21) = 1.74, *p* = 0.048, Hedge g = 0.74). Slope values that characterize the accuracy of responses did not differ between conditions. However, the total number of timed nose-poke visits was reduced after clomipramine (t(21) = 3.29, *p* = 0.0035, Hedge g = 1.40). At the same time, clomipramine did not affect the number of omissions, nor premature ones (Table 1). However, as total activity decreased under clomipramine, we also examined the percentage of premature responses and omission trials in relation to total timed responses. The percentage of premature responses did not change under clomipramine (zval: −0.6655, *p* = 0.51), while the percentage of omission trials significantly increased with clomipramine compared to the saline condition (t(21) = −4.55, *p* = 1.74 × 10^−4^, Hedge g = 1.94). To confirm that the effect of clomipramine on time perception and total activity was unrelated, we examined Pearson’s correlation regarding the difference between D50 and both total activity in the saline and clomipramine conditions as well as in omission trials (i.e., (D50 saline—D50 clomipramine) vs. (Total activity saline—Total activity clomipramine) and (Omission saline—Omissions clomipramine). We did not find any correlation of time perception changes with neither total activity (R(20) = 0.09, *p* = 0.69) nor omission (R(20) = −0.18, *p* = 0.43) effects.

## 4. Discussion

In the current study, we, for the first time, employed the IntelliCage system to study interval timing in rats and showed its promises in this regard. We anticipate that it can be a potential extension for the cognitive function testing in various preclinical studies, e.g., on testing drugs related to depression.

The advantage of the task is related to its ecological validity as rats live their own lives, as there is no need to move animals from their home cage and place them into experimental chambers. Moreover, there is substantial shortening of the training period as learning appears constantly and for a group of rats simultaneously compared to more traditional approaches, where animals are studied one by one. For example, Wistar rats in our experiment learned the task in about 1 week of training, while in the previous experiments with a similar interval bisection paradigm, about 10–40 sessions conducted in separate days were needed for each rat [47,48,49]. In the study where animals have to learn a longer duration, learning time is even greater. For example, in the study of [27], animals learned to discriminate 5 vs. 20 s and sixty 20 min sessions were required for each rat. A similar situation is found for the learning DRL task: to reach a stable baseline performance in this task with interresponse time within 10 s, it generally takes at least three weeks of daily training (30 min per day) for a rat (e.g., [50,51]). Learning DRL for 72 s takes much longer, e.g., more than 15 weeks with a rather complicated training schedule [33,34].

By applying this animal-friendly and resource-efficient approach, we found that in the baseline saline condition, D50 was slightly smaller than the objective bisection point, confirming the previous findings of time representation shrinkage obtained in humans [4,22,23]. Moreover, rats under acute clomipramine treatment shift their interval bisection point towards larger values, indicating their higher preference for the choice of short-duration response, corresponding to what was reported by Bizot in a standard and not automatized setting, including the comparable effect size (Hedge g = 1) [27]. As the currently perceived duration under the clomipramine condition is compared to those stored in memory under baseline conditions, this pattern of the result can be interpreted within the lossy integrator for the time model as an increase in internal time speed after acute clomipramine treatment and corresponds with the link of increased serotonergic functioning with an increase in internal time speed. This link can also be derived from previous studies both in animals and humans (see recent systematic review [6]). Thus, this approach provides the opportunity for the direct translation of the results between humans and laboratory animals.

We also have to point out that clomipramine decreased total activity and increased the percentage of omission trials in the animals, which generally corresponds with previous findings [27,52]. However, these changes were not correlated with changes in D50, which indicated the internal clock speed. Also, neither the precision of timed response (measured as a slope of the psychometric function) nor the number of premature responses were affected. Considering these results, we can suggest independence of changes in the internal clock speed from the accompanying changes in other motor and cognitive functions under acute clomipramine treatment. Noteworthily, in humans, a recent meta-analysis suggested a positive effect of antidepressants, including SRIs, on processing speed, executive function, attention, and memory but specifically for depressed persons, as no effects were confirmed for the non-depressed neurotypical controls [53]. Here, our rat sample should be considered as neurotypical; thus, no effect of general cognitive functioning is expected, which seems to be not fully confirmed.

Though clomipramine is a tricyclic drug and functions as both a serotonin and norepinephrine reuptake inhibitor, our research focused primarily on its effects on the serotonergic system. This focus is justified for several reasons. First, clomipramine demonstrates a stronger affinity for the serotonin transporter (SERT) than for norepinephrine transporters, making its serotonergic effects the dominant mechanism at the doses used [54]. Second, it was shown in rats that clomipramine used at a similar dosage (namely, 5 mg/kg) primarily influences serotonin levels without significantly affecting norepinephrine transmission [55]. At the same time, some noradrenergic effects might be suggested when interpreting the current results. In particular, we can speculate that the decrease in total activity might be attributed to it as previous studies consistently reported the decrease in total activity in various behavioral tests after treatment with selective norepinephrine reuptake inhibitors [35,56].

Several studies have explored the effects of clomipramine on 5-HT modulation in the rat brain, showing that it generally has minimal impact on extracellular serotonin levels in most brain regions, except for the raphe nuclei. Specifically, one found that clomipramine could reduce whole-brain 5-HIAA levels by up to 30%, while 5-HT levels remained largely unchanged (e.g., within cortex and hippocampus), regardless of acute or chronic administration [57]. In the raphe nuclei, acute administration has been observed to increase serotonin levels nearly threefold [58]. Additionally, clomipramine has been found to reduce regional cerebral metabolic rates for glucose, an indicator of neuronal activity, particularly in brain areas dense with 5-HT reuptake sites, such as the raphe nuclei and superior colliculus [59]. These findings suggest that the behavioral changes observed following clomipramine treatment might be attributed to alterations in raphe nuclei neural activity induced by 5-HT regulation. While norepinephrine undoubtedly contributes to clomipramine’s overall pharmacological profile, e.g., by reducing total activity, the change in interval timing is rather attributed to serotonergic functioning within the framework of the current study. Future research may expand on this work by disentangling the relative contributions of norepinephrine and serotonin to clomipramine’s behavioral effects.

We assume that the provided experimental design can enrich the behavioral arrays employed to study animals’ cognitive profile. Time perception is the basic cognitive function that is disturbed in a different psychopathology, such as major depression and schizophrenia [2,12,60,61]; in neurodegenerative diseases, such as Parkinson’s disease [2,62,63,64]; and in neurodevelopmental disorders, such as ASD [65] and ADHD [66]. It can be implicated in impulsive behavior, in changes in the elderly [67], and under stress [40,68,69]. Thus, introduced in the current study, the animal-friendly and resource-efficient experimental protocol to examine interval timing might have great potential for application to a wide range of fundamental and practical problems. It has potential to monitor the dynamics of the treatment effect and characterize the different animal models of psychopathology.

### Limitations and Future Directions

The side of reward responses were not counterbalanced across groups. However, the psychometric curve suggests that there was no response bias in the studied group of animals. Nonetheless, this issue should be controlled in future studies. As the goal of the study was to demonstrate feasibility to study the effect of drugs on time perception in rats using the IntelliCage system, we employed only one SRI and at only one dose. Future studies have to extend this research into several doses as well as examine the effect of other drugs both enhancing and attenuating serotonergic activity. It might also be relevant to examine dynamics of the antidepressants’ effects, which is also possible in the IntelliCage system.

Noteworthily, while we find general correspondence between human and animal studies, we have to point out the clear distinction between duration discrimination tasks where durations of two stimuli presented in a pair are compared and the interval bisection task, where the comparison is made based on the previously acquired duration representation and duration that are classified as long or short. While this interval bisection task is also employed for humans (e.g., [70]), this task has several disadvantages compared to the duration discrimination task, which can be considered as less confounded by other cognitive processes such as memory and categorization. To the best of our knowledge, such a paradigm with pairs of duration to compare was never employed in rodents, which we consider as an unfortunate oversight. We assume that rats are able to learn this task and that this is a promising approach to employ in a future study with the IntelliCage system. It might allow the examination of the “genuine” interval timing purified from potential confounders, such as relation to the memory representation formed previously. In particular, assessment of relative differences in the durations should be more sensitive to the effect of interest, especially when the longer durations are used. This type of paradigm modification will allow a more direct approach for the examination of chronic antidepressants’ effects as we do not need to be concerned about the recalibration of memory representation that could occur within a prolonged time of drug administration. We also have to warn about the generalization of the acute effect shown in our study to the chronic effect of an SRI, which is more common in human clinical practice, as acute and chronic SRI effects on cognitive and emotional functioning might be different [53,71].

There is also substantial research that has to be conducted before the proposed experimental design can be employed in preclinical trials, which include the examination of sensitivity and specificity of the test’s results in different psychopathological animal models in comparison to other methods used. In particular, it should be examined together with DRL for testing antidepressant effects.

## 5. Conclusions

To sum up, in this brief research report, we confirm the effect of clomipramine on time perception and present an animal-friendly and resource-efficient experimental paradigm to assess interval timing that can be applicable to clinical research on depression and potentially other psychopathology, where this function can be disturbed.

## Figures and Tables

**Table 1:** Mean (std) and ranges of measures obtained in IBT for Wistar rats (*n* = 22).

	D50	Slope	Total Activity	Omissions	Premature Responses
Saline	2.18 (0.55) 1.32–3.48	0.74 (0.35) 0.18–1.34	220 (123) 66–539	35 (20) 15–85	43 (38) 4–149
Clomipramine	2.42 (0.59) 1.63–3.60	0.80 (0.37) 0.01–1.39	115 (81) 39–369	30 (25) 10–140	29 (28) 5–113

## Data Availability

The scripts used in the current study can be downloaded at: https://github.com/RebikAnastasiya/IHNA-N-RAS-Parser-for-IntelliCage-TSE-custom-temporal-bisection-task-program; https://github.com/Kafuir/rat_temporal_bisection/blob/main/mice.py.
